# Predictors of successful diversion of cats and dogs away from animal shelter intake: Analysis of data from a self-rehoming website

**DOI:** 10.1017/awf.2023.8

**Published:** 2023-02-16

**Authors:** Lexis H Ly, Alexandra Protopopova

**Affiliations:** Animal Welfare Program, Faculty of Land and Food Systems, University of British Columbia, 2357 Main Mall, Vancouver, BC, Canada, V6T 1Z4

**Keywords:** animal adoption, animal sheltering, animal welfare, capacity for care, companion animals, intake diversion

## Abstract

As animals experience distress in animal shelters, leaders call for increased efforts to divert intake of companion animals away from shelters. One novel intake diversion strategy is supported self-rehoming, where owners find new homes for their animals without surrendering to a physical shelter. This study aimed to identify predictors of successful diversion of animals through the AdoptaPet.com ‘Rehome’ online platform. Data for dogs (n = 100,342) and cats (n = 48,484) were analysed through logistic regression to assess the association of animal- and owner-related factors and outcome. Overall, 87.1% of dogs and 85.7% of cats were successfully diverted from animal shelters, out of which, 37.8% of dogs and 35.3% of cats were kept by their original owner. Multiple animal-related factors predicted increased odds of diversion (e.g. younger, smaller). Dog and cat owners who set a longer rehoming deadline (i.e. > 8 weeks) were over twice as likely to keep or adopt out their animal. Dog owners who surrendered for owner-related reasons had increased odds of diversion in comparison to animal behaviour issues. We conclude that online-supported, self-rehoming platforms provide pet owners with an alternative to relinquishment that may reduce the intake of animals to shelters; however, owners with animals that are not preferred by adopters may have to decide whether to keep their animal or relinquish their animal to a shelter or rescue. These results provide guidance for animal shelter professionals on the likelihood of successful diversion programmes given certain animal and owner characteristics.

## Introduction

Animal shelters serve their communities through protecting animals and people from each other, supporting the human-animal relationship, and providing animal care (Zawistowski *et al.* 1998; Hurley 2022). However, animal shelters must remain at a reasonable number of animals inside its facility (i.e. capacity) to preserve resources, provide sufficient care for animals within the facility, and respond to community needs (Hobson *et al.* 2021). Capacity remains a critical issue in animal shelters (Horecka & Neal 2022), and many shelters are overcapacity due to a higher number of intakes compared to outcomes of the animals in their care. One way that shelters intake animals to facilities is through owner surrender, which makes up approximately 25–35% of shelter intake in North America (Humane Canada 2017; Shelter Animals Count 2022). While the circumstances leading to surrender are often multifaceted, recent research indicates that owner-related reasons (e.g. financial stress, difficulties finding pet-friendly housing) are more common primary reasons for surrender in comparison to animal behaviour issues (Alberthsen *et al.* 2016; Jensen *et al.* 2020; Eagan *et al.* 2022).

In response to concerns about capacity issues, animal shelters often make management decisions to reduce intake and increase adoption of animals in shelter facilities. For example, Capacity for Care (C4C) is a shelter management programme that recommends various procedures that aim to increase the speed at which animals can be adopted from the shelter, such as holding adoption specials and reducing the time it takes for an animal to be moved to the adoption floor (Hobson *et al.* 2021). As an additional measure to reduce capacity, many animal shelters have recently shifted toward managed intake, where animals are admitted on an appointment-basis to regulate the flow of animals (Hurley 2022). For animals that are not admitted to facilities immediately, animal shelters may use ‘intake diversion’ programmes, which are strategies that provide alternatives to intake, thereby removing the need for animals to enter the shelter altogether (Hurley 2022). Often, intake diversion interventions are based upon common reasons for surrender (Best Friends Animal Society 2019). For example, one common reason for which owners surrender animals to shelters is due to having ‘too many’ animals, supposedly the result of unwanted litters (Lambert *et al.* 2014; Eagan *et al.* 2022). To address this reason directly, animal shelters and veterinary clinics may offer low-cost or free spaying/neutering services in order to reduce the number of unwanted litters in the community (Frank & Carlisle-Frank 2007; White *et al.* 2010).

Intake diversion programmes can also provide an alternative for pet owners whose intake is not deemed urgent through shelter triage. One method to divert intake is through supported self-rehoming, where owners relinquish their animal directly to a new owner without surrendering their animal first to a shelter or rescue. The American Pet Products Association (2021) reported that 57% of pet owners said they would give their dog to a friend or relative if they could not care for the dog anymore. While bringing the animal to a shelter or rescue was the second most popular option, it was only reported by 16% of pet owners (American Pet Products Association 2021). Weiss and colleagues (2014) found that 85% of dog owners who had brought their animal to a shelter to surrender had tried to explore other options for rehoming before bringing the animal to the facility. Common methods of personal rehoming included giving the animal to family or friends, contacting a help line, and social media or online public advertisements (Weiss *et al.* 2014). Similarly, Digiacomo and colleagues (1998) interviewed people who previously relinquished animals, and found that 45% attempted to rehome their animals to friends, family, or through an online advertisement (Digiacomo *et al.* 1998), although owners were often unsuccessful in self-rehoming without additional support. This indicates that informal rehoming of animals does already occur, although support may be needed to improve the success of this diversion tactic.

In 2017, the website AdoptaPet.com (hereby referred to as Adopt a Pet) launched a supported self-rehoming programme called ‘Rehome.’ Adopt a Pet also functions as an online marketing tool for animal shelters and rescues to post available animals (like Petfinder.com). Adopt a Pet collects data similar to typical animal shelter data, including physical characteristics of the animal (e.g. breed, size), behaviour (e.g. good with dogs, good with children), and reason for rehoming. The platform also collects data on the outcome of the animal, including whether the animal was adopted or kept (i.e. diverted from animal shelters) or relinquished to a shelter/rescue after being posted for adoption on ‘Rehome’. As no previous research has evaluated dedicated supported self-rehoming programmes, our primary research aim was to understand which animal and owner characteristics predict increased odds of diversion from versus relinquishment to animal shelters, similar to previous research conducted within animal shelters. For example, previous studies found that adopted dogs were likely to be small (Brown *et al.* 2013; Siettou *et al.* 2014), light coloured (Posage *et al.* 1998; Lepper *et al.* 2002), and younger (Clevenger & Kass 2003; Normando *et al.* 2006). Dog behaviours during interactions, such as engaging in play and lying down next to the adopter, increased likelihood of adoption (Protopopova *et al.* 2014). Dogs and cats that are surrendered for owner-related reasons were more likely to be adopted than stray animals, while those surrendered for behavioural reasons were less likely to be adopted (Lepper *et al.* 2002). Based on evidence from previous research conducted in animal shelters, we hypothesised that younger, purebred, and smaller animals those that were labelled as having desirable behaviours (e.g. good with children, good with other dogs), and those that were surrendered for owner-related reasons would have greater odds of diversion from animal shelters.

Our additional interest was to understand the decisions that take place during the diversion process. Diverted animals were either adopted to new owners or kept by their original owner. To further understand the process of diversion from animal shelters, our secondary aim was to explore which animal and owner characteristics were associated with a change in the odds of the pet being kept by the original owner versus it being adopted to a new home. We did not have specific hypotheses regarding the impact of animal and owner characteristics on odds of adoption versus keeping the pet as the aim was exploratory in nature, due to a lack of research in the field of intake diversion.

## Materials and methods

### Data

All study protocols were reviewed and approved by the University of British Columbia’s Behavioural Research Ethics Board (H21-01729). The full data and R script used for analysis can be found at https://github.com/lexisly/rehome_diversion. The datasets used for both research questions originated from data of animals that were listed on Adopt a Pet Rehome website (‘Rehome’) from January 1, 2017, until May 21, 2021. The raw data file of animal listings contained 202,163 observations. From the original dataset, 3,435 animals were removed as they were uploaded by Adopt a Pet to detect bugs on the website. An additional eight animals were removed for having no species and no age. Listings that had multiple animals because they were a bonded pair (n = 4,086) or a litter of animals (n = 109) were removed, as postings only had fields to enter information about a single animal. Animals with duplicate listings which were posted on the same day and by the same owner were removed (n = 2,189), as the true listing could not be identified. An additional 8,297 animals were removed because they were uploaded and removed on the same day. The data were also restricted to those with a final outcome, meaning that the animal was adopted, kept by the owner, or relinquished to an animal shelter/rescue.

The final dataset used for descriptive analysis included 148,826 observations (Dog: n = 100,342; Cat: n = 48,484). The data were separated by species for the statistical models. For both dogs and cats, the data collected from pet owners through Rehome included basic information such as sex (female = 0; male = 1), age (puppy/kitten, young, adult, senior), spay/neuter status (intact = 0, spayed/neutered = 1), whether the animal was microchipped (no = 0, yes = 1), and whether the animal was purebred (no = 0, yes = 1). For dogs only, the data also included the size (small, medium, large) and the breed of the dog. The primary reported dog breeds were organised into breed groups by one author (AP) loosely based on the American Kennel Club (AKC) categories (Terrier, Herding, Hound, Sporting, Toy, Working; dogs in the non-sporting category and non-AKC breeds were reassigned into categories based on the history and/or traditional use of the breed).

The data also included behavioural information about the animal, including whether the animal was house-trained (no = 0; yes = 1), and whether the animal was good with dogs, cats, and children (no = 0; yes = 1 for each). Pet owners could also indicate whether the animal had special needs (no = 0; yes = 1) or required an experienced adopter (no = 0; yes = 1). These statements were available to the owner to select without definitions.

From the pet profile, the number of photographs of the animal were included in the model (0, 1, 2, 3, 4). Pet owners could also indicate the period that they had to rehome their animal (1 week or less = short, 2 to 4 weeks = medium, 8 weeks = long, no deadline = none) and a reason for rehoming their animal (animal behaviour issue, cost issue, abandoned or found, housing issue, human health issue, personal issue, none listed). These categories were available to the owner with no definitions. The first listed level in each variable was used as the reference level for the models. The owners also had an opportunity to write more about the animal, restrictions on adopters, and/or the circumstances behind the surrender in a textbox, but this text was not included in the present analysis.

### Analysis

All analyses were performed in R Studio version 2.3.492 (RStudio Team 2022). Descriptive analyses (e.g. proportion) were used to characterise the population of dogs and cats on the website by the provided variables. Prior to analysis, correlations between independent variables were tested using Phi correlation (for pairs of binary variables), Chi-squared Cramer’s *V* (for pairs with at least one nominal variable), and Spearman’s rank correlation (for pairs with at least one ordinal variable). All analyses were run separately by species (dog and cat).

For the first research question, we conducted a binary logistic regression model with the outcome of whether the animal was diverted (i.e. adopted or kept = 1) or relinquished to an animal shelter (= 0). The data were split into training (80%) and testing (20%) samples. Due to the unequal proportion of outcome classes, the training datasets were down-sampled such that the proportions of diverted and relinquished pets were equal (Dog: n = 10,322; Cat: n = 5,545).

We ran an additional opportunistic *post hoc* model for only the purebred dogs in the sample (n = 27,241) to test whether breed group was associated with a change in odds of diversion even for purebred dogs. The training dataset was down-sampled such that the proportion of diverted and relinquished pets were equal (n = 4,000).

For the second research question, the data were subset to remove animals that were relinquished to a shelter, so only those that were diverted remained. We performed a binary logistic regression with an outcome of being kept by their original owner (= 1) versus being rehomed (= 0). These data were split into training (80%) and testing (20%) samples and then down-sampled such that the proportions of adopted and kept pets were equal (Dog: n = 52,886; Cat: n = 23,488). Final regression models were selected using backwards elimination based on the Akaike Information Criterion (AIC).

## Results

### Descriptive analysis

In total, 148,826 animal records were used for analysis. The majority were dogs (67.4%), while the rest were cats (32.6%). For dogs (n = 100,342), 12.9% (n = 12,902) were relinquished to a shelter or rescue and the rest diverted (out of the total dog profiles, 54.2% [n = 54,387] were adopted, 32.9% [n = 33,053] were kept). For cats (n = 48,484), 14.3% (n = 6,931) were relinquished to a shelter or rescue and the rest diverted (out of the total cat profiles, 55.4% [n = 26,874] were adopted, 30.3% [n = 14,679] were kept).

The most common reported primary dog breeds in the sample were American Pit Bull Terrier (APBT; 11.3%, n = 11,582), Labrador Retriever (11.2%, n = 11,470), German Shepherd Dog (7.3%, n = 7,487), Chihuahua (6.2%, n = 6,322), and Husky (4.6%, 4,726). APBTs comprised the majority (58.3%) of the Terrier breed group, while Labrador Retrievers comprised the majority (66.3%) of the Sporting breed group. Across all dog breeds, the mean (± SD) percentage of purebred dogs across all breeds was 35.8 (± 19.2)%. For the most commonly reported primary dog breeds, percentage of dogs reported as purebred was 25.4% (n = 2,943) for APBT, 17.9% for Labrador Retriever (n = 2,055), 32.5% for German Shepherd Dog (n = 2,435), 38.0% for Husky (n = 1,796), and 18.9% for Chihuahua (n = 1,198).

### Statistical analysis

The correlation analyses revealed three pairs of independent variables that were at least moderately (i.e. coefficient > 0.3) correlated and statistically significant. As dogs’ age group increased, the proportion of spayed/neutered dogs increased (Puppy = 41.5%, Young = 72.5%, Adult = 84.7%, Senior = 87.7%, *r* = 0.32; *P* < 0.001). Dog size and breed group were moderately associated (Cramer’s *V* = 0.48; *P* < 0.001). The majority (54.4%) of small dogs belonged to a Toy breed, medium-sized dogs were relatively well-represented across breed groups other than Toy breeds (Herding = 23.6%, Hound = 11.6%, Sporting = 20.2%, Terrier = 22.0%, Toy = 4.2%, Working = 18.4%), and large dogs were mostly Working breeds (33.9%), Sporting breeds (21.0%), Herding breeds (18.4%) and Terrier breeds (15.0%). For cats, age and spay/neuter status were also moderately associated (ɸ = 0.53; *P* < 0.001), with the percentage of spayed/neutered cats being 30.4% for kittens, 77.0% for young cats, 93.2% for adult cats, and 96.0% for senior cats. All independent variables were included in our initial models, as the Variance Inflation Factor scores did not indicate problems of severe multicollinearity.

#### Which dogs were diverted?

Within the cleaned data (n = 100,342), 87.1% (n = 87,440) of the dogs were diverted from animal shelters after being posted on ‘Rehome’, while the rest were relinquished to a shelter or rescue facility. All the variables that were originally entered into the model are shown in Table 1.

**Table 1.** Animal- and owner-related characteristics across the total sample (n = 100,342) of dogs listed on ‘Rehome’, the diverted population, and dogs that were adopted to a new home or kept by their original owner. The data are from dogs listed on ‘Rehome’ from January 1, 2017 to May 21, 2021

	Total		Diverted		Of which	
	N	%	n	%	Adopted (%)	Kept (%)
**Sex**						
Male	54,390	54.2%	47,435	87.2%	62%	38%
Female	45,952	45.8%	40,005	87.1%	62%	38%
**Age**						
Puppy	19,630	19.6%	17,443	88.9%	64%	36%
Young	43,512	43.4%	37,964	87.2%	63%	37%
Adult	32,125	32.0%	27,789	86.5%	61%	39%
Senior	5,075	5.1%	4,244	83.6%	61%	39%
**Good with cats**						
True	32,122	32.0%	28,577	89.0%	63%	37%
False	68,220	68.0%	58,863	86.3%	62%	38%
**Good with dogs**						
True	74,808	74.6%	65,889	88.1%	63%	37%
False	25,534	25.4%	21,551	84.4%	59%	41%
**Good with children**						
True	75,349	75.1%	66,504	88.3%	63%	37%
False	24,993	24.9%	20,936	83.8%	59%	41%
**House-trained**						
True	83,759	83.5%	73,231	87.4%	62%	38%
False	16,583	16.5%	14,209	85.7%	64%	36%
**Purebred**						
True	27,241	27.1%	24,741	90.8%	62%	38%
False	73,101	72.9%	62,699	85.8%	62%	38%
**Microchipped**						
True	47,582	47.4%	40,777	85.7%	61%	39%
False	52,760	52.6%	46,663	88.4%	63%	37%
**Needs experienced adopter**						
True	29,115	29.0%	24,949	85.7%	56%	44%
False	71,227	71.0%	62,491	87.7%	65%	35%
**Special needs**						
True	6,500	6.5%	5,407	83.2%	54%	46%
False	93,842	93.5%	82,033	87.4%	63%	37%
**Spay/neuter status**						
True	71,377	71.1%	61,712	86.5%	62%	38%
False	28,965	28.9%	25,728	88.8%	63%	37%
**Rehome deadline**						
Short	24,950	24.9%	20,448	82.0%	67%	33%
Medium	44,908	44.8%	39,094	87.1%	64%	36%
Long	17,593	17.5%	16,045	91.2%	58%	42%
None	12,891	12.8%	11,853	91.9%	55%	45%
**Photographs on profile**						
0	2,141	2.1%	1,880	87.8%	50%	50%
1	13,211	13.2%	11,502	87.1%	60%	40%
2	17,091	17.0%	14,867	87.0%	62%	38%
3	17,891	17.8%	15,624	87.3%	64%	36%
4	50,008	49.8%	43,567	87.1%	63%	37%
**Rehome reason**						
Behavioural/issues	17,379	17.3%	14,500	83.4%	55%	45%
Cost issues	4,163	4.1%	3,548	85.2%	55%	45%
Abandoned or found	8,108	8.1%	6,904	85.2%	69%	31%
Housing issues	23,615	23.5%	20,705	87.7%	65%	35%
Human health issues	8,574	8.5%	7,460	87.0%	64%	36%
Personal issues	36,049	35.9%	32,153	89.2%	62%	38%
None listed	2,454	2.4%	2,170	88.4%	75%	25%
**Dog size**						
Small	28,885	28.8%	25,929	89.8%	65%	35%
Medium	45,638	45.5%	39,078	85.6%	62%	38%
Large	25,819	25.7%	22,433	86.9%	59%	41%
**Breed group**						
Terrier	19,400	19.3%	16,499	85.0%	61%	39%
Herding	18,007	17.9%	15,723	87.3%	61%	39%
Hound	10,138	10.1%	8,657	85.4%	62%	38%
Sporting	16,880	16.8%	14,644	86.8%	62%	38%
Toy	17,828	17.8%	16,159	90.6%	64%	36%
Working	18,089	18.0%	15,758	87.1%	62%	38%

**Figure 1.**
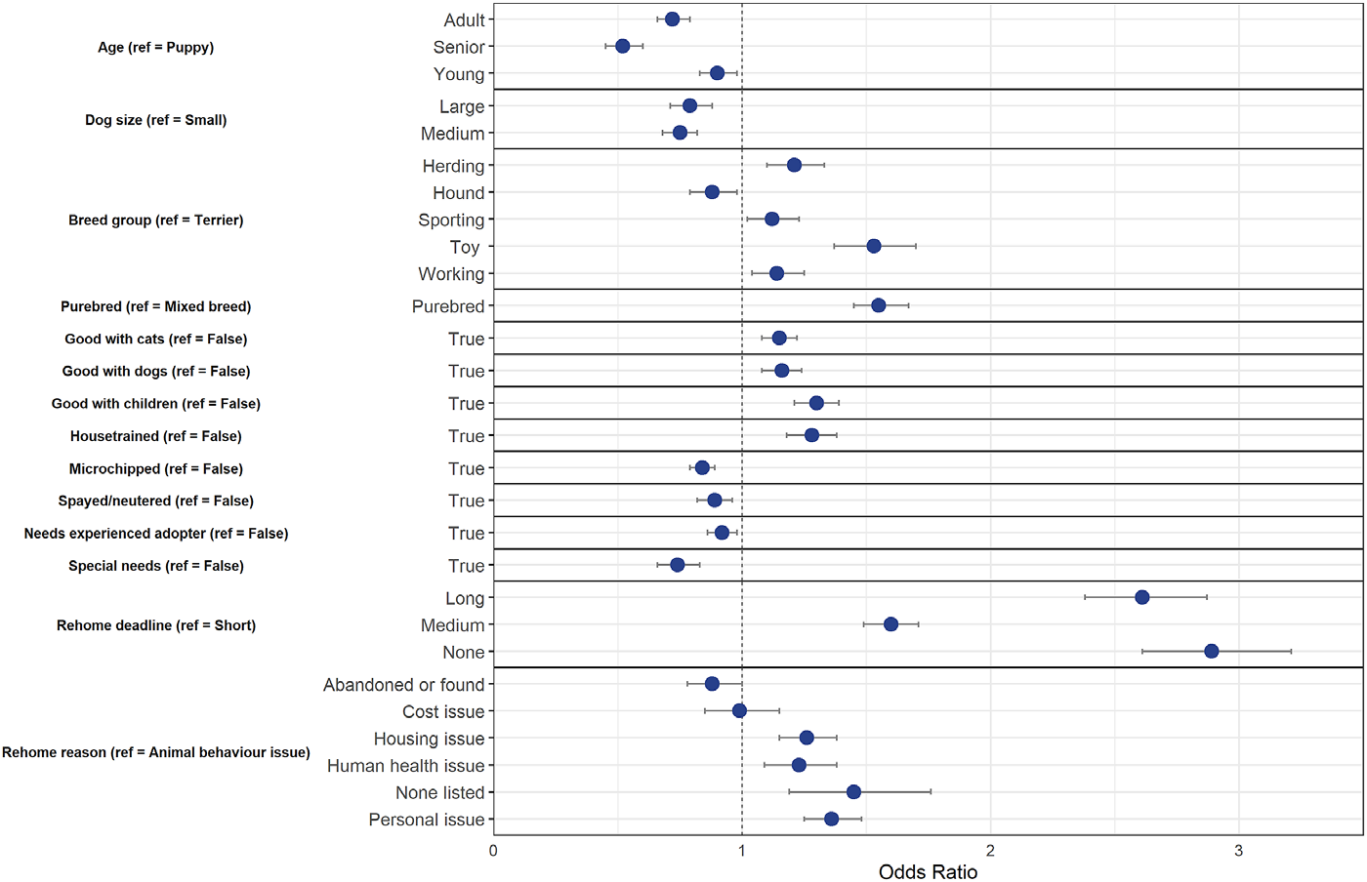
Associations between dog and owner characteristics and whether dogs were diverted (= 1) or relinquished to animal shelters (= 0) after being posted on an online-supported, self-rehoming website (n = 10,322). Data are presented by odds ratios and their 95% confidence interval (error bars); *P* < 0.05 when the 95% CI does not cross the vertical dotted line.

The final model using the down-sampled data (n = 10,322) contained 14 variables that were associated with a change in odds of diversion from animal shelters following backwards, step-wise elimination. The final model correctly classified 60.4% of the cases (95% CI = 59.8–61.1%) in the testing dataset. Figure 1 shows the final model variables; although within each of these variables, not all variable levels were statistically significant.

Several physical characteristics of dogs on ‘Rehome’ predicted a change in odds of diversion from animal shelters. As dogs increased in age group, the odds of diversion decreased in comparison to puppies by 10% for young dogs (95% CI = 0.83–0.98), by 28% for adult dogs (95% CI = 0.66–0.79), and by 48% for senior dogs (95% CI = 0.48–0.60). Both medium and large dogs had decreased odds of diversion by 0.75 (95% CI = 0.68–0.82) and 0.79 times (95% CI = 0.71–0.88), respectively, in comparison to small dogs. In comparison to Terrier breed dogs, Working (OR = 1.13, CI = 1.04–1.25), Sporting (OR = 1.12, 95% CI = 1.02–1.23), Herding (OR = 1.21, 95% CI = 1.10–1.33), and Toy breed (OR = 1.53, 95% CI = 1.37–1.70) dogs had increased odds of being diverted from an animal shelter. Dogs that were a Hound breed showed 0.88 times decreased odds of being diverted (95% CI = 0.79–0.98). Purebred dogs had 1.56 times greater odds of being diverted in comparison to mixed breed dogs (95% CI = 1.45–1.67).

The *post hoc* model revealed that breed groups also impacted odds of diversion even for purebred dogs. However, in comparison to Terrier dogs, only Sporting (OR = 1.35, 95% CI = 1.07–1.71) and Toy breed (OR–1.52, 95% CI =1.22–1.89) dogs had increased odds of diversion.

Behaviour and health status also predicted a change in odds of diversion for dogs. Dogs that were labelled good with cats, other dogs, and children had increased odds of diversion by 1.14 (95% CI = 1.08–1.22), 1.16 (1.08–1.24), and 1.30 times (1.21–1.39), respectively. Similarly, odds of diversion for dogs that were house-trained were 1.28 times greater in comparison to non-house-trained dogs (95% CI = 1.18–1.38). Dogs that were microchipped and spayed/neutered had 0.84 (95% CI = 0.79–0.89) and 0.89 times (0.82–0.96), respectively, decreased odds of diversion from animal shelters. Dogs that were labelled as needing an experienced adopter and dogs that were labelled as special needs had decreased odds of being diverted from animal shelters by 0.92 (95% CI = 0.86–0.98) and 0.74 times (95% CI = 0.66–0.83), respectively.

Owner circumstance, such as the deadline to rehome and the reason for rehoming, affected the odds of diversion for dogs. As the rehome deadline increased, dogs had increasingly greater odds of being diverted from animal shelters. Odds of diversion increased by 1.60 times for those with medium deadlines (95% CI = 1.49–1.71), 2.6 times for those with long deadlines (95% CI = 2.38–1.87), and 2.89 times for those with no deadline (95% CI = 2.61–3.21) in comparison to dogs with short rehome deadlines. In comparison to rehoming for animal behaviour issues, dogs that were rehomed for housing (OR = 1.45, 95% CI = 1.15–1.38), human health (OR = 1.23, 95% CI = 1.09–1.38) or personal issues (OR = 1.36, 95% CI = 1.25–1.48) had greater odds of diversion from animal shelters.

#### Which cats were diverted?

Within the cleaned data (n = 48,484), 85.7% (n = 41,553) of the cats were diverted, while the rest were relinquished to a shelter or rescue facility. All the variables that were originally entered into the model are shown by final outcome in Table 2.

**Table 2.** Animal- and owner-related characteristics across the total sample (n = 48,484) of cats listed on ‘Rehome’, the diverted population, and cats that were adopted to a new home or kept by their original owner. The data are from cats listed on ‘Rehome’ from January 1, 2017 to May 21, 2021

	Total		Diverted			
	n	%	n	%	Adopted (%)	Kept (%)
**Sex**						
Male	23,490	48.4%	20,202	86.0%	64%	36%
Female	24,994	51.6%	21,351	85.4%	65%	35%
**Age**						
Kittens	12,458	25.7%	10,949	87.9%	71%	29%
Young	15,704	32.4%	13,494	85.9%	63%	37%
Adult	17,971	37.1%	15,179	84.5%	63%	37%
Senior	2,351	4.8%	1,931	82.1%	59%	41%
**Good with cats**						
True	30,782	63.5%	26,571	86.3%	66%	34%
False	17,702	36.5%	14,982	84.6%	63%	37%
**Good with dogs**						
True	19,163	39.5%	16,698	87.1%	66%	34%
False	29,321	60.5%	24,855	84.8%	64%	36%
**Good with children**						
True	30,806	63.5%	26,722	86.7%	66%	34%
False	17,678	36.5%	14,831	83.9%	63%	37%
**House-trained**						
True	44,217	91.2%	37,916	85.7%	64%	36%
False	4,267	8.8%	3,637	85.2%	71%	29%
**Purebred**						
True	3,480	7.2%	3,190	91.7%	65%	35%
False	45,004	92.8%	38,363	85.2%	65%	35%
**Microchipped**						
True	15,307	31.6%	12,854	84.0%	63%	37%
False	33,177	68.4%	28,699	86.5%	65%	35%
**Needs experienced adopter**						
True	6,497	13.4%	5,498	84.6%	55%	45%
False	41,987	86.6%	36,055	85.9%	66%	34%
**Special needs**						
True	2,245	4.6%	1,837	81.8%	51%	49%
False	46,239	95.4%	39,716	85.9%	65%	35%
**Spay/neuter status**						
True	34,887	72.0%	29,765	85.3%	64%	36%
False	13,597	28.0%	11,788	86.7%	67%	33%
**Rehome deadline**						
Short	10,752	22.2%	8,716	81.1%	67%	33%
Medium	22,738	46.9%	19,376	85.2%	66%	34%
Long	9,045	18.7%	8,124	89.8%	63%	37%
None	5,949	12.3%	5,337	89.7%	58%	42%
**Photographs on profile**						
0	1,078	2.2%	912	84.6%	51%	49%
1	7,200	14.9%	6,036	83.8%	64%	36%
2	8,260	17.0%	7,019	85.0%	64%	36%
3	8,347	17.2%	7,139	85.5%	66%	34%
4	23,599	48.7%	20,447	86.6%	65%	35%
**Rehome reason**						
Behavioural/issues	7,459	15.4%	6,327	84.8%	55%	45%
Cost issues	2,228	4.6%	1,924	86.4%	56%	44%
Abandoned or found	10,052	20.7%	8,585	85.4%	71%	29%
Housing issues	12,722	26.2%	10,867	85.4%	65%	35%
Human health issues	7,581	15.6%	6,485	85.5%	67%	33%
Personal issues	7,373	15.2%	6,434	87.3%	64%	36%
None listed	1,069	2.2%	931	87.1%	73%	27%

**Figure 2.**
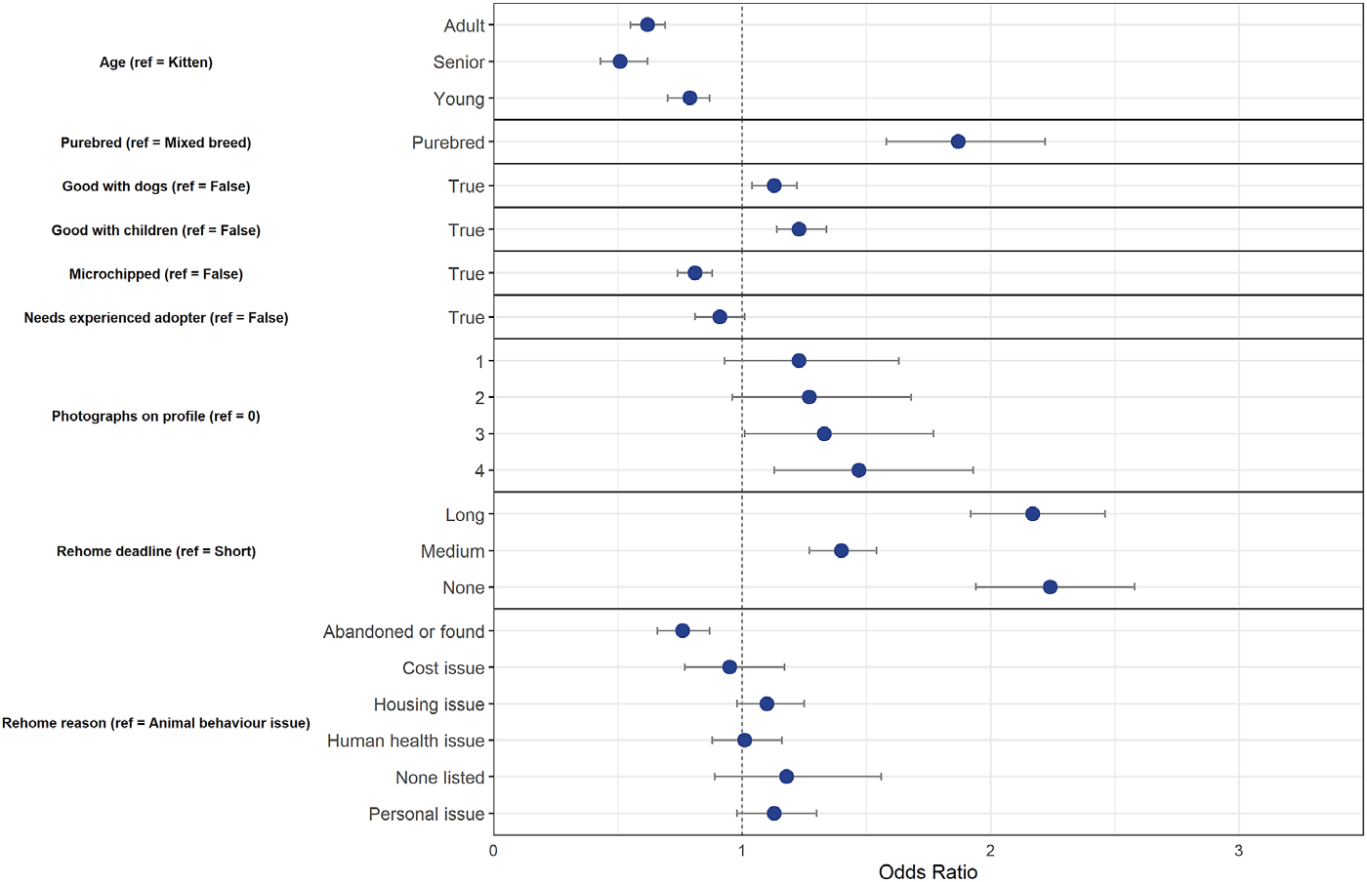
Associations between cat and owner characteristics and whether cats were diverted (= 1) or relinquished to animal shelters (= 0) after being posted on an online-supported, self-rehoming website (n = 5,545). Data are presented by odds ratios and their 95% confidence interval (error bars); *P* < 0.05 when the 95% CI does not cross the vertical dotted line.

The final model using the down-sampled data (n = 5,545) contained nine variables that were associated with a change in odds of diversion from animal shelters (Figure 2). The final model correctly classified 56.1% of the cases (95% CI = 55.1–57.1%) in the testing dataset. Figure 2 shows the final model variables; although within each of these variables, not all variable levels were statistically significant.

Similarly to dogs, physical characteristics of cats such as age and being purebred influenced the outcome of cats posted on ‘Rehome.’ As age groups increased, odds of diversion from animal shelters decreased by 21% for young cats (95% CI = 0.70–0.87), by 38% for adult cats (95% CI = 0.55–0.69), and 49% for senior cats (95% CI = 0.43–0.62). Purebred cats had 1.87 times greater likelihood of diversion in comparison to mixed breed cats (95% CI = 1.58–2.22).

While being good with other cats did not change the outcome, cats that were good with dogs and children had 1.13 (95% CI = 1.04–1.22) and 1.23 times (95% CI = 1.14–1.34) increased odds of being adopted or kept, respectively. Cats that were microchipped had 0.81 times less chance of being diverted from animal shelters compared to non-microchipped cats (95% CI = 0.74–0.88).

While the number of photographs on dogs’ profiles was not statistically significant, the number on cats’ profiles was relevant in predicting outcome — cats who had three (OR = 1.33, 95% CI = 1.01–1.77) or four photographs (OR = 1.47, 95% CI = 1.13–1.93) had greater odds of being diverted in comparison to having none on the profile.

Similarly to dogs, as the rehome deadline increased, the odds of diversion increased by 1.40 times for those with a medium deadline (95% CI = 1.26–1.54), by 2.17 for a long deadline (1.92–2.46), and by 2.24 for those with no deadline (95% CI = 1.94–2.58) to rehome their cat. Unlike for dogs, the only reason for rehoming that was statistically significant was for cats who were abandoned or found — these individuals had decreased odds of being diverted from animal shelters (OR = 0.76, 95% CI = 0.66–0.87).

#### What happened to diverted dogs?

**Figure 3.**
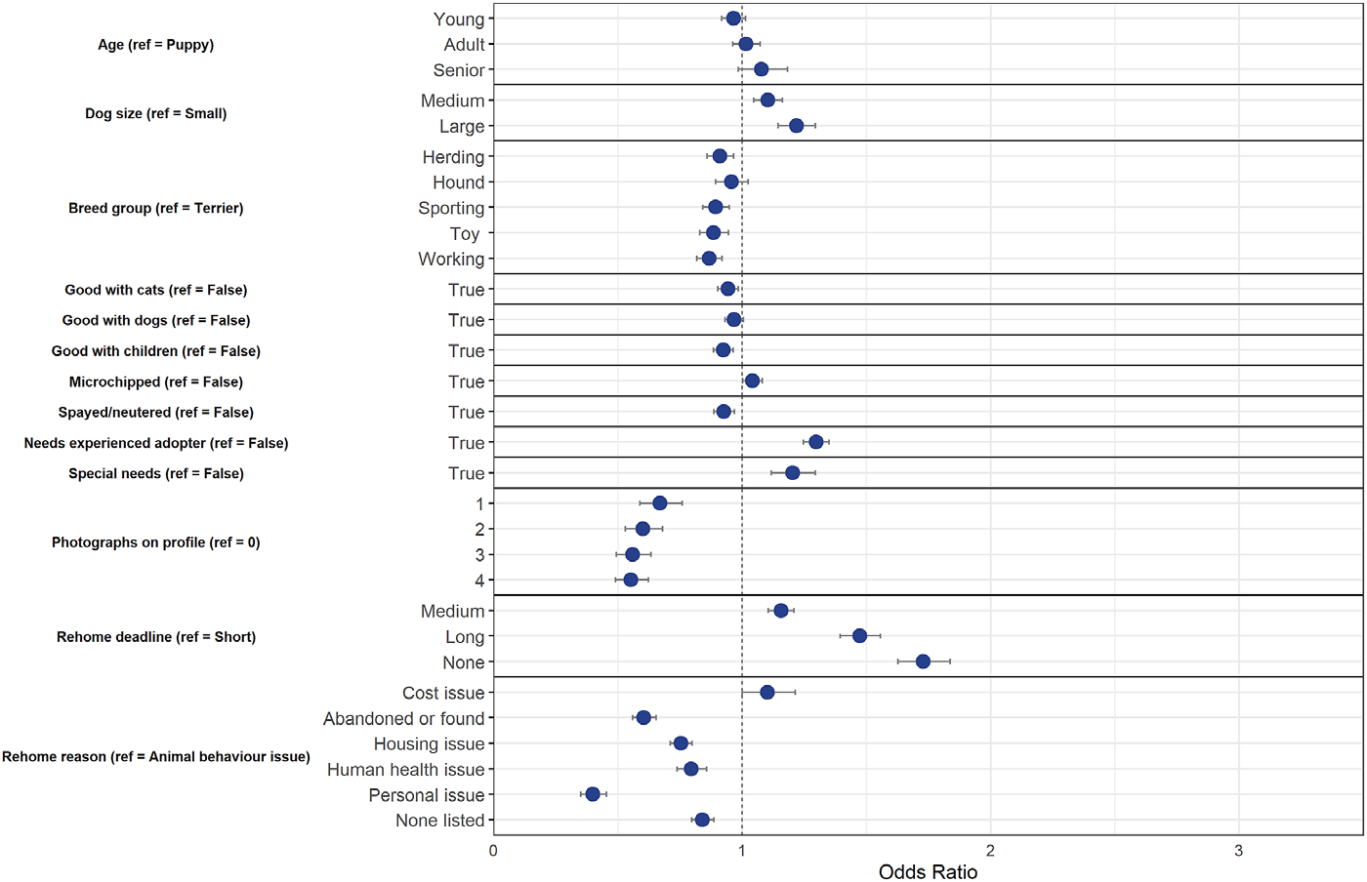
Associations between dog and owner characteristics and whether dogs were kept by their original owner (= 1) or adopted to a new home (= 0) after being posted on an online-supported, self-rehoming website (n = 52,886). Data are presented by odds ratios and their 95% confidence interval (error bars); *P* < 0.05 when the 95% CI does not cross the vertical dotted line.

Out of dogs that were not relinquished to a shelter or rescue (n = 87,440), 37.8% (n = 33,053) were kept by their owners, while the remaining 62.2% (n = 54,387) were rehomed. The final model correctly classified 57.0% of the cases (95% CI = 56.3–57.8%) in the testing dataset. The final model using the down-sampled data (n = 52,886) contained 13 variables that were associated with a change in odds of keeping versus adopting dogs; although within each of these variables, not all variable levels were statistically significant (Figure 3).

Both medium- and large-sized dogs were more likely to be kept by their owner rather than rehomed, in comparison to small (medium OR = 1.16, 95% CI = 1.11–1.21; large OR = 1.22, 95% CI = 1.15–1.29) dogs. Herding, Sporting, Toy, and Working breed dogs had decreased odds of being kept by their original owner in comparison to Terrier breeds by 0.91 (95% CI = 0.86–0.97), 0.89 (95% CI = 0.84 = 0.95), 0.87 (95% CI = 0.83–0.94), and 0.87 times (95% CI = 0.82–0.92), respectively.

Dogs that were good with cats or children had only slightly decreased odds of being kept in comparison to those that were not (good with cats OR = 0.94, 95% CI = 0.90–0.98; good with children OR = 0.92, 95% CI = 0.88–0.96). Dogs that were spayed or neutered had 7% decreased odds of being kept in comparison to intact dogs (OR = 93, 95% CI = 0.88–0.97). Dogs that needed an experienced adopter had 1.30 times greater odds of being kept by their owner (95% CI = 1.25–1.35). Similarly, special needs dogs had 1.20 times increased odds of being kept by their owner (95% CI = 1.12–1.29).

As the number of photographs on a dogs’ profile increased, odds of an owner keeping their dog decreased in comparison to having no photographs on the profile (one OR = 0.67, 95% CI = 0.60–0.76; two OR = 0.60, 95% CI = 0.53–0.68; three OR = 0.56, 95% CI = 0.49–0.63; four photographs OR = 0.55, 95% CI = 0.49–0.62).

As the owner’s rehome deadline increased from a short deadline, the odds of keeping the dog increased by 1.15 times for a medium rehoming deadline (95% CI = 1.11–1.21), by 1.47 times for a long rehoming deadline (95% CI = 1.39–1.56), and by 1.73 times for a those with no rehoming deadline (95% CI = 1.63–1.84). In comparison to animal behaviour issues, dogs that were being rehomed for numerous owner-related issues had lower odds of being kept. Odds of being kept decreased by 0.40 times for dogs whose rehoming reason was the owner’s personal issues (95% CI = 0.35–0.45). Dogs that were originally abandoned to the current owner or found had 0.60 times less chance of being kept by their owner (95% CI = 0.56–0.65). Odds of being kept decreased by 0.75 times for dogs rehomed for housing-related issues (95% CI = 0.71–0.80) and by 0.80 times for human-health issues (95% CI = 0.74–0.86). Dogs rehomed for cost-related issues had only marginally increased odds of being kept (OR = 1.10, 95% CI = 1.00–1.21).

#### What happened to diverted cats?

**Figure 4.**
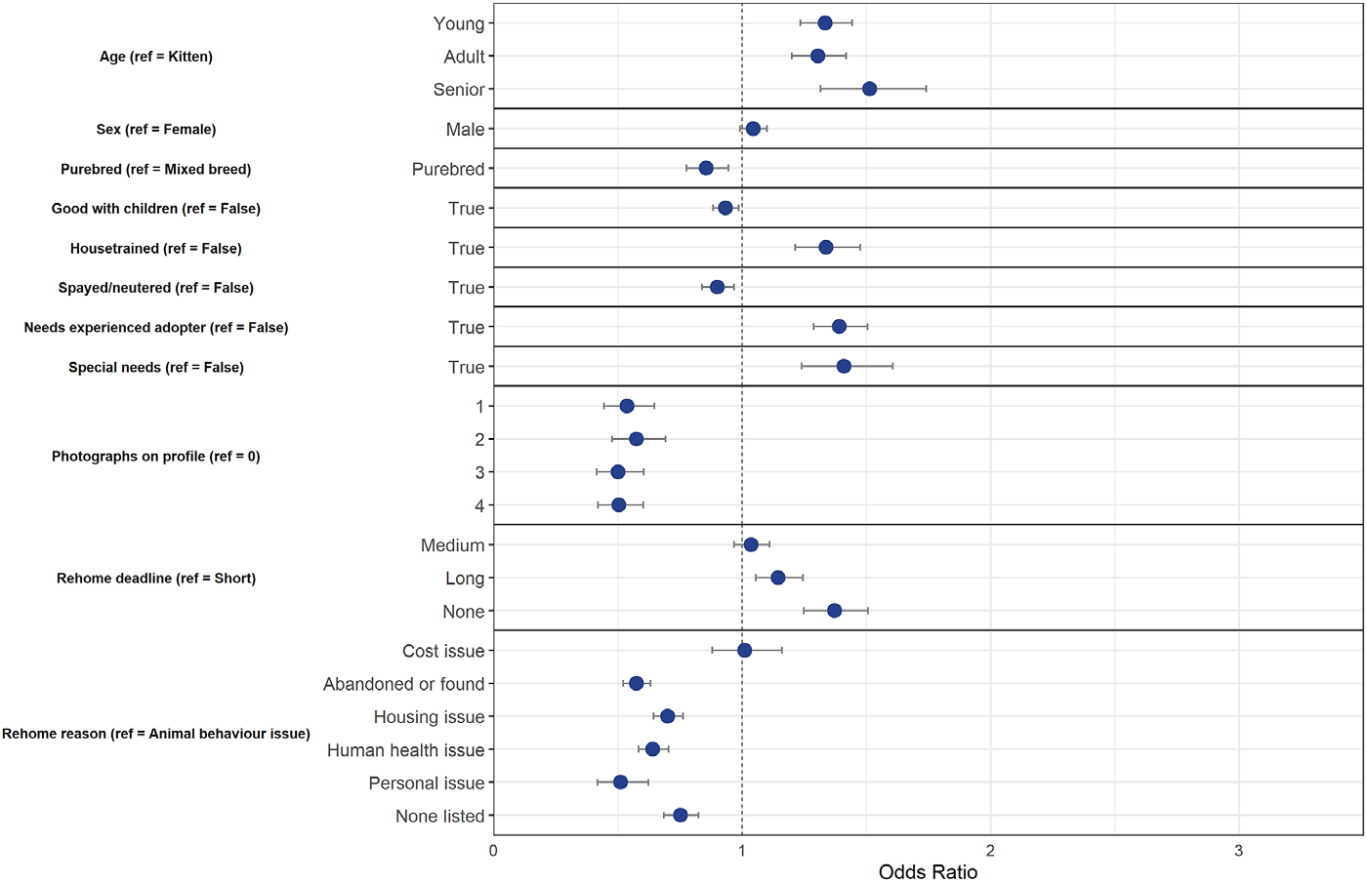
Associations between cat and owner characteristics and whether cats were kept by their original owner (= 1) or adopted to a new home (= 0) after being posted on an online-supported, self-rehoming website (n = 23,488). Data are presented by odds ratios and their 95% confidence interval (error bars); *P* < 0.05 when the 95% CI does not cross the vertical dotted line.

Out of cats that were not relinquished to a shelter/rescue (n = 41,553), 35.3% (n = 14,679) were kept by their original owners, while the remaining 64.4% (n = 26,874) were adopted to a new home. The final model using the down-sampled data (n = 23,488) contained eleven variables that were associated with a change in odds of the owner keeping their cat; although within each of these variables, not all variable levels were statistically significant (Figure 4). The final model correctly classified 58.3% of the cases (95% CI = 57.3–59.4%) in the testing dataset.

Cats in all three older age groups had increased odds of being kept by their owners in comparison to kittens (young OR = 1.33, 95% CI = 1.23–1.44; adult OR = 1.30, 95% CI = 1.20–1.42; senior OR = 1.51, 95% CI = 1.31–1.74). Purebred cats had 0.86 times decreased odds of being kept in comparison to mixed breed cats (95% CI = 0.87–0.94).

Cats that were good with children had slightly decreased odds of being kept by their original owner (OR = 0.93, 95% CI = 0.88–0.98). Cats that needed an experienced adopter had 1.39 times greater odds of being kept (95% CI = 1.29–1.51), while cats that had special needs had 1.41 times greater odds of being kept (95% CI = 1.24–1.61). Also, odds of being kept were 1.33 times greater for house-trained cats (95% CI = 1.21–1.48), but the odds of being kept were decreased by 0.90 times for spayed or neutered cats in comparison to those intact (95% CI = 0.84–0.97).

As the number of photographs on a cats’ profile increased, odds of a cat being kept decreased in comparison to having none on the profile (one OR = 0.54, 95% CI = 0.44–0.65; two OR = 0.57, 95% CI = 0.44–0.65; three OR = 0.50, 95% CI 0.48–0.69; four photographs OR = 0.50, 95% CI = 0.42–0.60).

The odds of being kept by their owner did not differ significantly from those who had a medium rehome deadline in comparison to a short deadline. However, cats with a long deadline or no deadline for rehoming were more 1.15 (95% CI = 1.05–1.24) and 1.37 times (95% CI = 1.25–1.51) more likely to be kept, respectively. Similarly to dogs, cats that were rehomed due to owner-related reasons (other than cost-related issues) were less likely to be kept in comparison to animal behaviour issues. Odds of being kept decreased by 0.70 times for cats who were rehomed due to housing-related issues (95% CI = 0.64–0.76), by 0.64 times for human health issues (95% CI =0.58–0.70), and by 0.51 times for personal issues (95% CI = 0.42–0.62). Also, cats that were originally abandoned to the owner or found were 0.75 times less likely to be kept in comparison to those rehomed due to animal behaviour issues (95% CI = 0.68–0.83).

## Discussion

Approximately 85% of both cats and dogs were adopted to a new home or kept by their owners after being posted online, suggesting that supported self-rehoming could be a useful tool to reduce the intake of animals to shelter and rescue facilities. In our study, the remaining 15% of animals were still relinquished to a shelter, although the odds of relinquishment differed based on animal- and owner-related characteristics. While previous research has investigated sources of acquisition of companion animals (e.g. Freiwald *et al.* 2014; Weiss *et al.* 2015; Bir *et al.* 2017), adoption through supported self-rehoming has not been recognised as a separate acquisition source. However, Weiss and colleagues (2015) found that the most common method of rehoming pets was to a friend or family member (37%), while an additional 11% were given to someone that was not previously known to the pet owner. In comparison, 36% of relinquishing pet owners still brought their animal to a shelter or rescue (Weiss *et al.* 2015). Bringing supported self-rehoming to online pet adoption platforms, such as Adopt a Pet, that potential adopters have already visited when searching for animals (Weiss *et al.* 2012; Workman & Hoffman 2015), may further promote the use of supported self-rehoming as a way of diverting animals from shelters.

The population of dogs and cats posted on ‘Rehome’ was generally younger, smaller, and had a higher proportion of morphologically preferred dog breeds and purebred cats in comparison to animal shelter populations described in previous literature. In our study, 5.1% of dogs and 4.8% of cats were seniors. Previous studies reported that the proportion of senior dogs in shelters ranged from 7–18%, while the senior cat population proportion ranged from 8–14% (New Jr *et al.* 1999; Scarlett *et al.* 1999; Hawes *et al.* 2020). Our study population had a similar proportion of purebred dogs (27.1%) in comparison to older in-shelter studies, which ranged from 24–30% (New Jr *et al.* 1999; New *et al.* 2000). However, while most older studies are based on visual identification and owner reports, Gunter and colleagues (2018) found that only 5% of shelter dogs had only one majority breed through genetic testing. Our study also found a slightly higher proportion of purebred cats (7.2%) in comparison to previous reports (2–6%; New Jr *et al.* 1999; New *et al.* 2000; Shore & Girrens 2001). Hawes and colleagues (2020) reported a higher proportion of Terrier (27.5 versus 19.3%) and Herding (21.6 versus 17.9%) dogs, while our sample had a much higher proportion of Toy (17.8 versus 4.9%) and Hound (10.1 versus 5.9%) dogs. Similarly, our study also reported a higher proportion of small dogs (28.8 versus 15.7%; Hawes *et al.* 2020). Given that we found that animals of a young age, purebred status, certain breed types, and small size to be more likely to be diverted from animal shelters, this suggests that animal shelter data represent a particular sub-sample of all owner-surrendered pets.

### Predictors of diversion and retention

In line with our hypothesis, morphological characteristics that are preferred by adopters in shelter settings were also important in predicting the type of diversion through self-rehoming (i.e. remaining with the original owner or entering a new home). Morphological features are prioritised by adopters, compared to behaviour, when potential adopters initially evaluate shelter dogs in-person (Protopopova *et al.* 2012; Weiss *et al.* 2012). Indeed, this may be even more true for adopters searching for pets on the internet since there is no possible physical interaction with the animals. Research in animal shelters as well as on online adoption websites similarly found that likelihood of adoption for dogs and cats decreased with increased age (Lepper *et al.* 2002; Workman & Hoffman 2015). Similarly, previous research found that purebred dogs were 1.43 times more likely to be adopted rather than euthanased (Lepper *et al.* 2002). Lepper and colleagues did not evaluate whether purebred cats had an increased likelihood of adoption; however, rare breeds, such as Siamese, were more likely to be adopted in comparison to domestic shorthair cats. More generally, when pet owners were asked why they did not adopt their animal from a shelter, 35% cited the desire for a purebred animal (Maddalena *et al.* 2012), indicating that potential adopters value purebred animals, similar to our study.

Congruent with the results of our study, previous research found larger dogs to be less likely to be adopted from animal shelters (Lepper *et al.* 2002; Brown *et al.* 2013; Siettou *et al.* 2014). Physically, individual breeds within each group may differ greatly, particularly when the animal is mixed breed. However, we also found that, even within purebred dogs, Sporting and Toy dog breeds still had greater odds of diversion. Preference for particular breeds and smaller dogs indicates that appearance is important in the decision to adopt an animal through online-supported, self-rehoming programmes. In addition to the morphological differences seen across dog breeds, breed labels may influence potential adopters’ perceptions of behaviour or attractiveness of dogs. In our study, APBTs were the most common dog breed posted on ‘Rehome’, in addition, this breed comprised the majority of the Terrier breed group. Similarly, in animal shelters, pit bull-type dogs are often the most prevalent breed-label available for adoption (Protopopova *et al.* 2012; Voith *et al.* 2013). Breed labelling in animal shelters often relies upon owner reports or visual identification of staff, which is often inaccurate in comparison to DNA analysis, particularly for pit bull-type dogs (Voith *et al.* 2009). While in the present study, the impact of breed on the odds of diversion was likely a mix between perception of dog breed labels and physical characteristics, breed labelling by owners may be even more important on websites like Adopt a Pet, where prospective adopters can filter their search based on dog breed.

While previous in-shelter research used measures such as interactions with adopters and in-kennel location and behaviours to evaluate whether behaviour mattered to adopters (Luescher & Tyson Medlock 2009; Protopopova *et al.* 2012; Grant & Warrior 2019), our study used owner-reported behavioural indicators. Animal shelters and online adoption websites may include tags such as ‘good with children’ and ‘needs experienced adopter’ when displaying available animals to inform potential adopters about the animals’ behaviour. Indeed, in agreement with our hypothesis, dogs and cats that were good with dogs and children had increased odds of diversion. For dogs, being good with cats also increased odds of diversion. In a survey of potential adopters, approximately three-quarters rated behaviour with people as an important factor in dog and cat adoption, while approximately one-quarter rated behaviour with other animals as important (Weiss *et al.* 2012). Behavioural issues, particularly incompatibility with humans or other animals, are among the most common reasons for animals being returned to shelters (Mondelli *et al.* 2004; Gates *et al.* 2018; Scott *et al.* 2018; Hawes *et al.* 2020). However, the tags on ‘Rehome’ may also suggest undesirable behaviours or matching issues between the animals and a potential adopter’s new home (Protopopova & Bollen 2022). Indeed, shelter dogs labelled as ‘good with children’ had increased adoption rates in one US shelter (Luescher & Medlock 2009). This is further corroborated by the present study, as dogs and cats that were labelled as needing an ‘experienced adopter’ had greater odds of being relinquished to an animal shelter after being posted on ‘Rehome.’ Also, dogs that were labelled as ‘special needs’ had lower odds of diversion, perhaps because the label suggests difficult cost or medical requirements for the pet. Previous authors have suggested that staff refrain from adding these descriptors to the profiles of animals, and instead discuss them during the adoption counselling phase (Protopopova & Bollen 2022); although this may be more difficult to implement with individual relinquishing owners in comparison to trained adoption counsellors in shelter organisations.

Dogs rehomed due to behavioural issues were more likely to end up being eventually surrendered to an animal shelter. Similar to our results, Lepper and colleagues (2002) found that dogs that were relinquished for behavioural reasons were less likely to be adopted in comparison to stray dogs, although dogs relinquished for owner-related reasons were more likely to be adopted. Public members traditionally hold the belief that shelter dogs may differ behaviourally in comparison to dogs sourced elsewhere (Patronek *et al.* 2022). A survey from the Republic of Ireland found that 68% of dog owners who adopted their animal from a shelter reported at least one behavioural issue, with the most common being fearfulness (Wells & Hepper 2000). However, research of owned dogs initially sourced from shelters, breeders, pet stores, and other sources shows that approximately 40–85% of the surveyed population reports that their dog has at least one behavioural issue (Voith *et al.* 1992; Kobelt *et al.* 2003; Blackwell *et al.* 2008; Scott *et al.* 2018). Our findings may indicate that dogs with owner-reported behavioural issues may have less success in adoption through online-supported, self-rehoming, and thus shelter facilities may serve as a safety net for such dogs. A rise in surrendered dogs with behavioural issues in shelter facilities may result in lack of adoption, increased length of stay (Normando *et al.* 2006; McGuire *et al.* 2021; Raudies *et al.* 2021), increased resource use to care for animals (Bradley & Rajendran 2021), and increased euthanasia due to behavioural reasons (Caras 1993; Pegram *et al.* 2021). However, further research is needed to understand whether dogs entering shelters have more prevalent or prominent behavioural problems that may require additional care and resources when surrendered to animal shelters. Animal shelter organisations can consider increasing the resources that are available for behavioural support of animals to accommodate dogs with behavioural concerns.

In contrast, behaviour was not as important in cat diversion. The lack of relationship between owner-related reasons and odds of diversion may indicate that cat adopters do not have preference for cats that were surrendered for particular reasons; however, our other results suggest that adopters do value certain behavioural traits (i.e. good with dogs, good with children, does not need an experienced adopter). The rehome reason selected by the owner is not displayed on the pets’ online profile, although owners may choose to disclose the reason in their pets’ profile biography. It may be possible that cat owners do not describe the behavioural issues in their pets’ biographies as often as dog owners do, or that behavioural issues that lead to surrender in cats may not be as deterring as behavioural issues in dogs. However, the online rehoming platform did not specify what behavioural issues led to relinquishment and the present study did not assess the biographies of pet profiles.

In contrast, dogs and cats labelled with ‘good’ behavioural characteristics were less likely to be kept (i.e. more likely to be rehomed). In addition, those that required an experienced adopter or had special needs had greater odds of being kept by their original owner. Relatedly, our results showed that for both species, all owner-related reasons decreased odds of being kept in comparison to rehoming due to behavioural issues, with the exception being cost-related issues for cats. Again, these results may be due to adopter preference for animals that are owner surrendered that do not have reported behavioural issues (Wells & Hepper 1992). Perhaps in addition, our results suggest that pet owners may feel that some behavioural issues are not strong enough to warrant the decision to relinquish or rehome the animal. Previous research has found that the majority of pet owners report that their animal engages in at least one behaviour that may be undesirable, such as leash pulling, hyperactivity, inappropriate elimination, and aggression (Patronek *et al.* 1996; Blackwell *et al.* 2008; Casey *et al.* 2014). Scott and colleagues (2018) found that, despite over 50% of dog owners reporting undesirable behaviours, almost all owners reported being satisfied with their pets’ behaviour. Indeed, many pet owners keep pets despite reporting behaviours that owners deem problematic (Voith *et al.* 2009).

Microchipped dogs and cats and spayed/neutered dogs had decreased odds of diversion from animal shelters. On the contrary, some studies have found that dogs and cats that are spayed/neutered are preferred by adopters to intact ones (Lepper *et al.* 2002). However, this discrepancy may be due to a greater proportion of intact and non-microchipped animals being younger in our study, as potential adopters largely prefer to adopt younger pets (Lepper *et al.* 2002; Normando *et al.* 2006; Weiss *et al.* 2012). In animal shelters, spay/neuter status may not be as important to predict adoption outcomes, as most animal shelters in North America spay or neuter their animals prior to adoption in order to reduce the risk of unwanted litters (Protopopova & Gunter 2017). However, spaying/neutering prior to adoption is not necessarily the case for animals adopted through online-supported, self-rehoming programmes, which leaves the choice to adopt an intact animal up to the individual. A recent survey of the US public found that 74% agreed that spaying and neutering pets was the right thing to do; however, 52% agreed that spay/neuter surgeries are expensive, and 34% agreed that the surgery can be dangerous (Glasser 2021). Online-supported, self-rehoming platforms may give those who prefer to adopt an intact animal a source to adopt, although further research is needed to understand whether these methods of private rehoming increases risk of unwanted litters.

For cats, but not dogs, the number of photographs on the profile impacted odds of diversion from animal shelters, with those that had three or four photographs having a greater likelihood of diversion in comparison to those with none. Previous research using online adoption websites also found that probability of adoption increased as the number of photographs on the pets’ profile increased (Lampe & Witte 2015; Markowitz 2020); although these studies included dogs, cats, and other species (e.g. horses, rabbits, small animals) in their analysis. The difference between dogs and cats may be due to potential adopters’ values when adopting each species. For example, while appearance was rated the most important factor when adopting dogs, behaviour with people was the most important for cats (Weiss *et al.* 2012). People may value additional photographs when viewing cats, as multiple photographs may give adopters a better judgement of behaviour or personality.

Owners who do not have time to engage in the rehoming process were at greater risk of relinquishing their animal to a shelter or rescue. As the deadline for rehoming was extended, the odds of diversion increased greatly. Pet owners often struggle with the decision to relinquish their pet for a prolonged period, which may lead pet owners to visit the shelter to surrender at the last possible moment — when the decision to surrender has been solidified (Digiacomo *et al.* 1998). Also, the decision to bring an animal to a shelter facility may be influenced by the pet owners’ attitudes toward shelters or euthanasia (Digiacomo *et al.* 1998; Lund *et al.* 2010; Martin *et al.* 2021). Our study similarly indicates that those who make the decision to post their animals with a short deadline to rehome are more likely to relinquish their animal to a shelter or rescue facility, indicating that relinquishment may be a last resort. Shelter staff could consider including supported self-rehoming resources on their websites or on their social media, where pet owners often look before they visit the shelter to surrender their animal (Workman & Hoffman 2015). Additionally, shelter staff may consider suggesting online-supported, self-rehoming as an alternative to surrender for those who contact them if they do not have a short timeline for rehoming their animal. Further research may consider investigating the decision-making process for pet owners who are considering relinquishment to determine the most meaningful methods to communicating alternatives to surrender such as supported self-rehoming.

Indeed, increased time to rehome the animal increased odds of the owner ultimately keeping their animal. This result may indicate that those who post their animal with a longer rehome deadline may have more time to access services that enable them to keep their animal. Weiss and colleagues (2014) found that over half of dog owners who brought their pet to a shelter for relinquishment considered their decision for a month or more before bringing their animal to shelter. As such, those who are in the earlier stages of the relinquishment decision-making process may be more successful candidates for pet retention programmes; however, further research is needed to understand how best to connect owners with these programmes, as research indicates that pet owners do not approach shelter and rescue organisations until later in the decision-making process (Digiacomo *et al.* 1998).

In situations where the owner did not have direct responsibility for the animal, the animal was not likely to be successfully diverted. Cats that were originally abandoned to or found by the current owner had decreased odds of diversion. One Australian shelter study reported that common reasons for cats being surrendered to shelters were that the cat was not theirs, they were concerned for the cat, or they thought the cat would be better off in the shelter (Zito *et al.* 2016). Indeed, in our study, it is possible that an owner may feel less personal responsibility towards the animal in comparison to a pet that has been owned by them for some time, leading them to bring the animal to the shelter quicker.

### Limitations and future directions

It is not fully clear whether the outcome of diversion is driven more by potential adopters’ desire to adopt or the original owners’ desire to keep their animal. Additionally, based on the current data, we cannot conclude whether an owner choosing to keep their pet led to eventual relinquishment or if they were truly able to retain their pet in their own family. Weiss and colleagues (2014) found that over half of surveyed relinquishing dog owners stated that a form of assistance (e.g. low-cost training, veterinary care, temporary pet-friendly housing) may have helped them retain their dog. In this same study, most relinquishing pet owners demonstrated a strong attachment to their pets, indicating that had resources been available to help them keep their dog, they would have considered using them (Weiss *et al.* 2014). As such, supported self-rehoming programmes could consider directing all pet owners with resources to encourage retention of their animals before the use of rehoming services. However, resources for owner support may be community specific (Weiss *et al.* 2014), rather than more generally applicable to all pet owners.

Although animals with the less desirable morphological and behavioural characteristics had the greatest odds of being relinquished, animals surrendered to shelters only represented about 15% of the total population posted on ‘Rehome.’ Further, animals entering shelters through owner surrender only represent an estimated 25–35% of animal shelter intake (Humane Canada 2017; Shelter Animals Count 2021; Rodriguez *et al.* 2022). As our sample only represents a small portion of the total shelter population, it is likely that animals entering through other means (e.g. stray, intake, humane officer investigation) may differ physically or behaviourally. Wells and Hepper (2000) investigated dogs that were adopted from shelters and found that stray animals were more likely to have owner-reported undesirable behaviours than surrendered animals. Within shelters, one study found that owner-surrendered cats showed more behavioural signs of stress in comparison to stray cats (Dybdall *et al.* 2007). While further research is required to understand differences in populations from various sources, the present study shows that the population of animals relinquished after being posted on supported self-rehoming websites are less preferred by adopters than those that are diverted.

The data collected by ‘Rehome’ relies on owner reports, which may lead to issues with consistency across animal characteristics. For example, the terms ‘needs experienced adopter’ and ‘special needs’ do not have any formal definition on the website. Previous studies that assessed owner-reported behaviour found that owners indicated a variety of behaviours that were considered problematic, including leash pulling, barking, and aggression to people or pets (New *et al.* 2000; Guy *et al.* 2001). While some owners feel that certain behaviours are incompatible with their lifestyle, others may not rate the same behaviours as problematic (Voith 2009). Grouping all behavioural incompatibilities into general categories may be detrimental to the adoption success of animals (Patronek *et al.* 2022). Further, behavioural indicators of pets posted on supported self-rehoming websites rely on subjective reports from relinquishing owners, which may not fully reflect the behaviours of animals in other environments. For example, cats who are labelled as ‘not good with dogs’ may be indoor cats that have not interacted with dogs. Stephen and Ledger (2007) surveyed owners who relinquished their dogs to shelters about their dogs’ behaviour and found that less than half of the behaviour ratings correlated to the responses of the dogs’ new owners. Similarly, owners report the singular reason for relinquishment on ‘Rehome’, although previous literature indicates that relinquishment reasons are multifaceted, often consisting of both animal- and owner-related reasons (Digiacomo *et al.* 1998). Future research should consider the consistency with which owners are reporting behavioural and health concerns and relinquishment reasons when posting their animal on supported self-rehoming websites, and whether these reports correspond to the animals’ welfare in a new environment.

As an additional benefit, online supported self-rehoming may reduce the emotional impact of surrendering an animal as it allows pet owners greater agency in the rehoming process, although this was not directly evaluated in the present study. Pet owners may undergo emotional distress when deciding to relinquish their animal, including internal conflict of whether their pets’ quality of life can be improved through rehoming (Buller & Ballantyne 2020). Pet owners who surrendered their animal to a shelter or rescue often struggle with the decision due to concerns of euthanasia in shelters (Digiacomo *et al.* 1998). Supported self-rehoming could allow pet owners to ensure that the animal is adopted to a new home and to even allow for the owner to select the adopter themselves, which may also increase agency and possibly reduce the emotional impact of relinquishment for pet owners, although further research is needed. However, in animal sheltering, one concern is that the subjectivity of traditionally restrictive adoption practices may allow for bias and discrimination against certain adopters (Best Friends Animal Society 2021; Maddie’s Fund 2021). In recent years, many animal shelters have introduced more ‘open’ adoption policies, such as the ‘Adopters Welcome’ programme (HumanePro 2020), which encourages the removal of restrictive adoption practices (e.g. home checks, age restrictions) in favour of conversation-based practices. Rehoming from one owner to another may subject potential adopters to bias or discrimination based on sociodemographic conditions, although further research would be required to support this hypothesis.

### Animal welfare implications

Companion animals in shelter facilities experience a variety of stressors, including unfamiliar surroundings, loud noises, lack of space, and insufficient social and environmental enrichment (Hennessy *et al.* 1997; Kry 2007; Ellis & Wells 2010; Scheifele *et al.* 2012). Supported self-rehoming may alleviate stress associated with shelter stays by removing the need to intake animals into these unfamiliar environments. However, given that those with less desirable characteristics had the greatest odds of being relinquished to animal shelters, organisations may end up with a larger than expected population of pets with less-preferred characteristics, which may lead to increased length of stay and resource use for organisations (Bradley & Rajendran 2021; McGuire *et al.* 2021). Understanding the pet and owner characteristics that lead to diversion versus shelter relinquishment can inform animal shelters of the population that may still enter their facilities.

## Conclusion

Our data showed that, on an online-supported, self-rehoming platform, various animal and owner characteristics influenced the odds of diversion from animal shelters. The results add to a growing body of literature that investigates methods to reduce intake of animals to shelter facilities. Identifying animal and owner characteristics that are associated with increased odds of diversion could inform animal shelter organisations of pets that may be more easily diverted through online-supported self-rehoming. Our analysis indicates that dogs and cats that are younger, purebred, are being surrendered for owner-related reasons, and do not have special needs or behavioural challenges have greater success in being diverted through supported self-rehoming. Also, owners who indicate an extended deadline for rehoming their animals should be diverted to an online-supported, self-rehoming platform. However, a small population are subsequently relinquished to shelter facilities after being posted on supported self-rehoming websites. Animals with behavioural challenges, including those who are being surrendered for behavioural issues, have greater odds of being surrendered to a shelter or rescue. As such, animal shelters may consider redistributing their resources to accommodate for behavioural challenges. In addition, understanding differences between animals that are adopted versus kept by their original owner could provide insight into demographics that may lead to retention. Overall, we conclude that online supported self-rehoming platforms provide pet owners with an alternative to relinquishment that may reduce the intake of animals to shelters.
